# Cascaded adaptive load frequency control for single area and double area power systems considering wind penetration

**DOI:** 10.1038/s41598-026-61216-z

**Published:** 2026-07-16

**Authors:** Ahmed EL-Bassiouny, Mahmoud A. Attia, Rizk Hamouda, Mohamed R. Hamouda

**Affiliations:** 1 Electrical Power and Machines Department Faculty of Engineering, Ain Shams University Cairo, Cairo, Egypt; 2 Independent Electricity System Operator, Toronto, ON Canada

**Keywords:** Load frequency control (LFC), Automatic generation control (AGC), Harmony search algorithm (HS), PI controller, PID controller, Adaptive fractional order PI controller (AFOPI), Energy science and technology, Engineering, Mathematics and computing

## Abstract

This study investigates the application of a cascaded adaptive controller for Load Frequency Control (LFC) in single-area and two-area power systems. The controller is a combination between adaptive PI controller and PID controller thus the term cascaded controller. The main objective is to evaluate the controller’s performance under various operating conditions through comparison with a conventional controller and adaptive controller. The case studies in the single-area power system shall be four case studies to be examined: (1) a conventional system without renewable energy integration, (2) a system with wind power introduced as a disturbance source, and (3) and (4) modified versions of the first two cases excluding time-delay effects. Similarly, the two-area power system is analyzed using four case studies, for the first and second scenarios, a static load change is applied independently to each area, while the third and fourth scenarios extend these cases by considering dynamic, time-varying load changes. For all scenarios, disturbances are introduced in one area, and their effects on tie-line power flow are analyzed. An optimization algorithm is utilized to determine the optimal gain parameters for each controller configuration. MATLAB/Simulink is employed for system simulation, and the system responses are assessed in the time domain. Simulation results demonstrate that the proposed cascaded adaptive controller exhibits superior performance and robustness, particularly in disturbance rejection and frequency stability enhancement. As there is an improvement in the system response by minimizing overshoot, reducing oscillations, and achieving faster settling times—outperforming traditional PI, standalone AFOPI, and PID controllers indicating that it can be utilized in different power systems.

## Introduction

Electrical power systems can generally be classified into several categories, ranging from conventional single-area systems to more complex multi-area systems interconnected through tie-lines. In recent years, power systems have undergone a significant transformation toward modern configurations that incorporate renewable energy sources. For reliable and efficient operation, a continuous balance between power generation and load demand must be maintained while considering disturbances, fluctuations, and transmission losses. System stability critically depends on maintaining frequency within its nominal range, as frequency is directly related to active power balance. Any mismatch caused by load variations, external disturbances, or changing operating conditions can result in frequency deviations, potentially driving the system from a stable state to an unstable state. Consequently, the primary role of Load Frequency Control (LFC) is to regulate the balance between generated power and load demand. LFC utilizes speed governors and control loops to detect generator speed deviations and restore equilibrium between mechanical and electrical torques, thereby ensuring proper control of system frequency and power output^1–8^. As reported in^9^, Proportional–Integral (PI) and Proportional–Integral–Derivative (PID) controllers are extensively used in Automatic Generation Control (AGC), which is often considered synonymous as LFC, due to the effectiveness of PI or PID in improving system stability and damping oscillations. More recently, advanced control approaches such as Integral Order (IO) and Fractional Order (FO) controllers have been introduced. Experimental studies indicate that FO controllers generally outperform IO controllers in terms of dynamic response and robustness. According to studies^10–15^, maintaining nominal system frequency and regulating tie-line power flow are essential requirements in multi-area power systems and this is achieved by driving the Area Control Error (ACE) to zero at steady state, which necessitates optimal tuning of controller parameters using advanced optimization techniques, such as the Harmony Search (HS) algorithm.

In this paper, a hybrid control strategy that integrates a cascaded control structure with an adaptive controller is proposed to exploit the advantages of both methodologies. The proposed systems are modeled and simulated using MATLAB/Simulink. Two different power system configurations are investigated. The first configuration is a single-area power system analyzed through four case studies. The first case considers the nominal operating condition of the system without wind power disturbances and without any time delay. The second case introduces a time delay of 2 s into the system, as adopted in^16^. To further evaluate the system robustness after incorporating the delay, additional tests are conducted by increasing the time delay to 4 s and 6 s. The third case study examines the impact of wind generation as a disturbance while including the time delay modeled according to^16^. Finally, the fourth case corresponds to the third scenario but excludes the time delay, thereby isolating the effect of wind power disturbances on system performance. The simulation results obtained for these cases are compared with those reported in^16,17^, where a conventional PI controller was employed, in order to assess the performance enhancement achieved by the proposed cascaded adaptive controller. The second configuration examined in this study is a two-area power system consisting of two interconnected single-area systems linked via tie-lines, enabling power exchange between the areas^18–20^. This system is also evaluated through four case studies. In the first and second scenarios, a load change is applied to only one area at a time, and the resulting frequency responses of both areas, along with the tie-line power flow, are analyzed. The third and fourth scenarios replicate the first two cases, respectively, but replace static load changes with dynamic, time-varying load disturbances. The simulation outcomes are compared with those presented in^18^, which employed a classical PID controller. This comparative analysis further demonstrates the effectiveness of the proposed cascaded adaptive control scheme in enhancing dynamic performance and maintaining frequency stability.

This paper is organized into the following sections. Part II presents a literature review. Part III details the proposed controller architecture, including gain selection and the governing equations. Part IV discusses the single and double area power systems, their parameters, and the results obtained. Then a comparative analysis is provided based on the various case studies. Part V concludes the paper, summarizing the findings and highlighting the effectiveness of the proposed control strategy.

## Literature review

Table 1 illustrates a summary regarding LFC in chronological progression for the single and multi-area power systems with a comparison between contribution and limitations.

**Table 1:** Literature review summary.

Reference	Controller used	System Type	Main Contribution	Observed Limitations
^4–7^	Classical PI/PID	Single- & multi-area	Simple and widely adopted controllers for frequency and tie-line regulation.	Limited robustness under nonlinearities, uncertainties, and time-varying disturbances.
	Fractional-Order PID (FOPID)	Single- & multi-area	Enhanced tuning flexibility and improved dynamic performance using fractional calculus.	Increased computational complexity compared to integer-order controllers.
^21,22^	Adaptive Fractional-Order PI (AFOPI)	Single- & multi-area	Improved adaptability, accuracy, and robustness under parameter variations.	Requires careful tuning of adaptive and fractional parameters.
^20–23^	Cascaded Control	Industrial & Power Systems	Faster disturbance rejection via inner–outer loop architecture.	Complex tuning and dependence on accurate system modeling.
^24^	SA–QI Optimized PID	Multi-area	Reduced frequency deviation and overshoot using hybrid optimization.	High computational burden limits real-time scalability.
^25^	Comprehensive LFC Review	Multi-area	Overview of classical, intelligent, and RES-based LFC techniques and smart grid challenges.	Broad scope limits in-depth evaluation of specific controllers.
^26^	Cascade P–P–FOPID	Multi-area	Reduced settling time and strong robustness to parameter uncertainties.	Increased structural and computational complexity.
^27^	Cascade PI–(1 + FOPID) (WHO)	Multi-area	Improved frequency response and robustness using WHO optimization.	Validation is limited to simulation studies.
^28–31^	Hybrid & Intelligent Controllers	Multi-area with High RES	Effective LFC under high renewable penetration and varying load conditions.	High design and computational complexity.

In this research, a proposed cascaded AFOPI controller shall be used in both single area power systems and double area power systems for faster response and enhanced stability.

## Load frequency control

### AFOPI controller

LFC is performed using controllers, and the most common controller is the classical PI and PID controllers. The PI controller output signal is generated by combining two components. The first component is the proportional term, which is the product of the error and the proportional gain, and the second component is the integral term, which is computed as the time integral of the error signal scaled by the integral gain. This structure enables the controller to provide immediate corrective action based on the current error and cumulative compensation to eliminate steady-state error over time^21^. The classic PI controller Transfer function is as follows from (2), where Kp is the proportional gain and Ki is the integral gain.

1 $$\:TF={K}_{p}+\:\frac{{K}_{i}}{S}$$

**Fig. 1:**
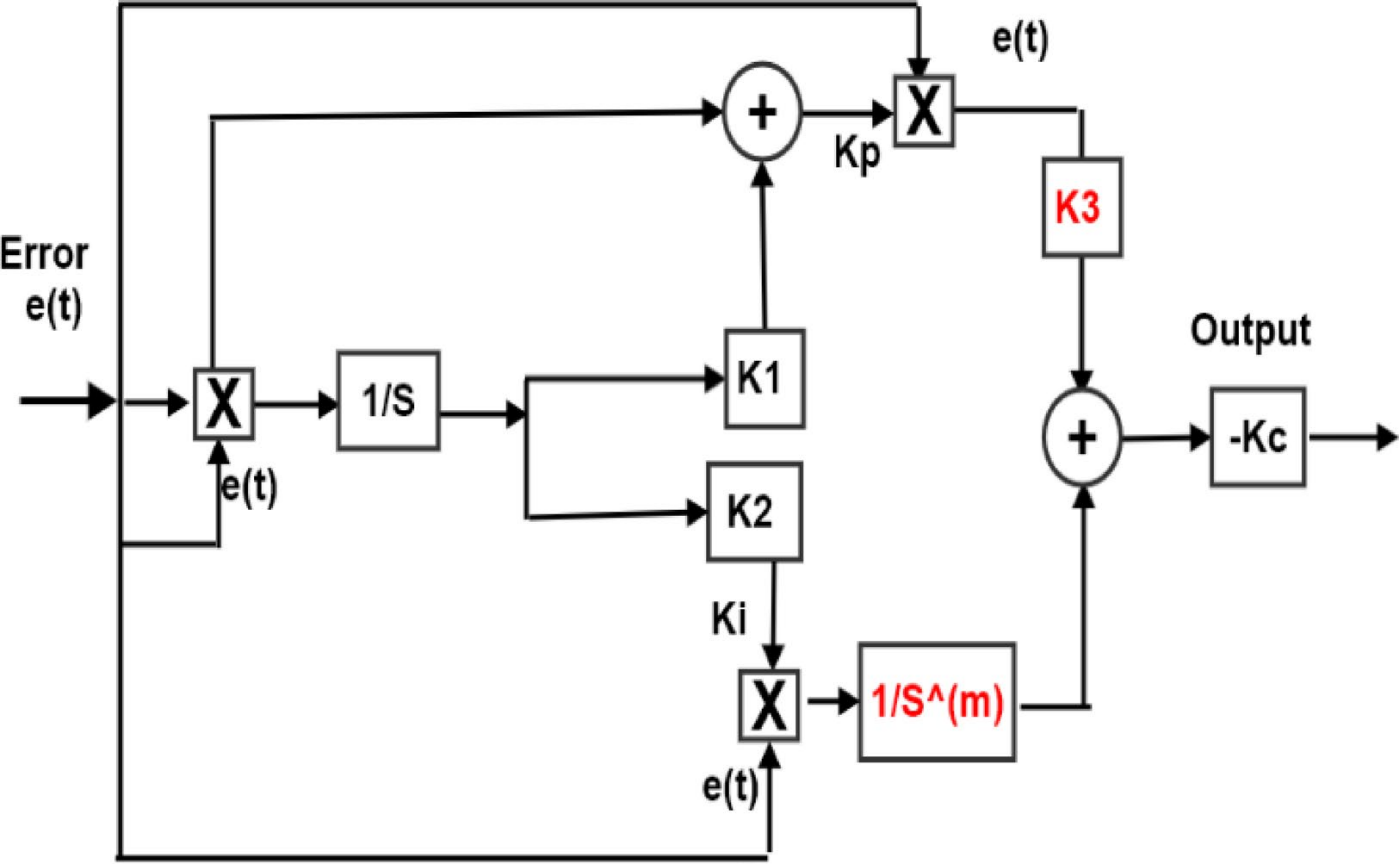
Adaptive fractional order PI controller.

Reference^17^ introduced a new Adaptive Fractional-Order PI (AFOPI) controller, which combines the features of an adaptive PI controller and a fractional-order PI controller. This hybrid approach offers advances over the classical PI controller, providing superior performance due to its self-adjusting gains and the inherent advantages of fractional-order control^32–37^. Figure 1 illustrates the block diagram of the Adaptive Fractional-Order PI controller (AFOPI). Equations (2), (3), and (4) represent the mathematical formulation of the AFOPI controller, as detailed in^36,37^. Optimizing the initials of such controllers has always presented a challenge. To address this, optimization techniques are employed, and in this research, the Harmony Search (HS) algorithm is used due to its simplicity and.

efficiency^37,38^.

2 $$\:\mathrm{K}\mathrm{p}=\mathrm{e}\mathrm{r}\mathrm{r}\mathrm{o}\mathrm{r}\mathrm{*}\mathrm{e}\mathrm{r}\mathrm{r}\mathrm{o}\mathrm{r}+\mathrm{K}1{\int\:}_{0}^{\mathrm{t}}\mathrm{e}\mathrm{r}\mathrm{r}\mathrm{o}\mathrm{r}\mathrm{*}\mathrm{e}\mathrm{r}\mathrm{r}\mathrm{o}\mathrm{r}\:\mathrm{d}\mathrm{t}\:$$

3 $$\:\mathrm{K}\mathrm{i}=\mathrm{K}2{\int\:}_{0}^{\mathrm{t}}\mathrm{e}\mathrm{r}\mathrm{r}\mathrm{o}\mathrm{r}\mathrm{*}\mathrm{e}\mathrm{r}\mathrm{r}\mathrm{o}\mathrm{r}\:\mathrm{d}\mathrm{t}\:$$

4 $$\:\mathrm{O}\mathrm{u}\mathrm{t}\mathrm{p}\mathrm{u}\mathrm{t}=-\mathrm{K}\mathrm{c}(\mathrm{K}3\mathrm{*}\mathrm{K}\mathrm{p}\left(\mathrm{t}\right)\mathrm{e}\mathrm{r}\mathrm{r}\mathrm{o}\mathrm{r}+\left(\frac{1}{{S}^{m}}\right)Ki\left(t\right)error\:$$

Where m is the integration order.

The initial parameters K1, K2, K3, and Kc are continuously adjusted in response to the error signal, with initialization for the adaptive controller achieved through the application of an optimization algorithm. M is the order of the fractional integrator. The objective function used for this optimization is the sum of the squared errors. The controller is termed “adaptive” because its parameters, including the proportional gain Kp and integral gain Ki are time-varying and dynamically adjusted based on the error signal.

### Harmony search algorithm

**Fig. 2:**
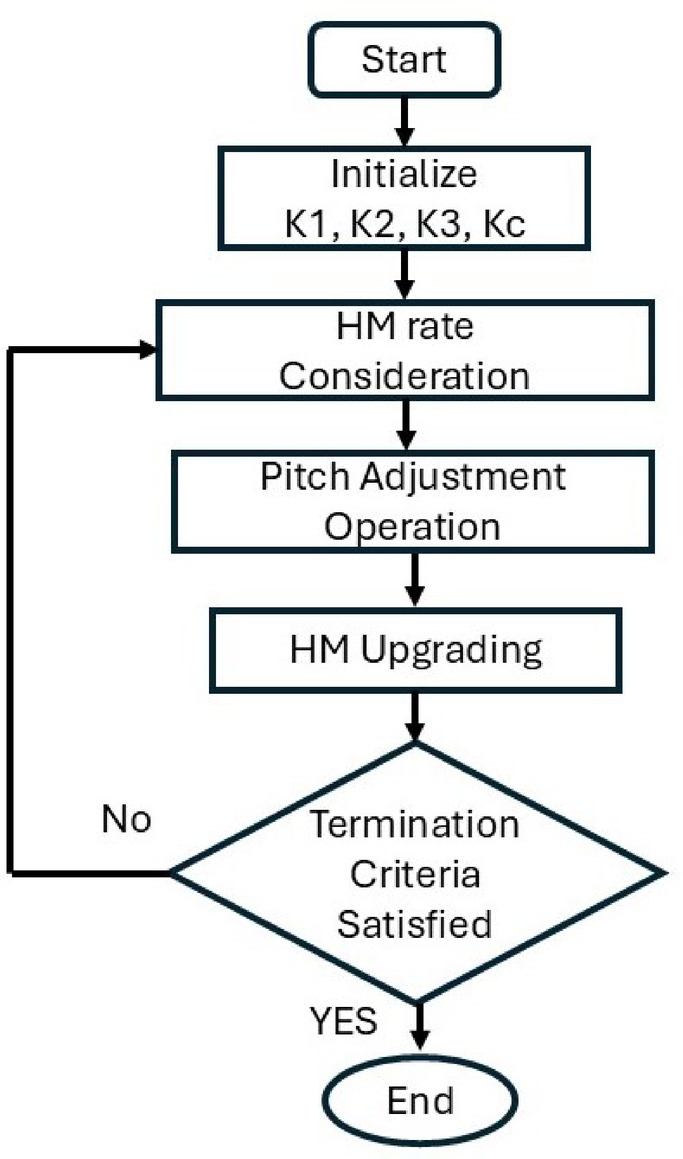
HS algorithm flow chart.

The Harmoney Search Algorithm (HS) is one of the optimization techniques used in LFC as it is simple and effective. HS implementation consists of four steps: (1) initializing the HSA Memory (HM), (2) improvising a new solution, (3) updating the HM through the condition if the new harmony yields a better objective function value, it replaces the worst harmony to improve overall solution quality in HM, and (4) repeating the process until termination criteria is met by reaching the optimization solution. Those steps are started by initializing the boundaries of the required variables and in our case the initial parameters of the controllers^17,28^. Figure 2 shows the flow chart of the HS algorithm. Boundaries or ranges of the gains are set based on trial and error.

### Double area objective function

The main objective function for the double area power system is to minimize the Area Control Error (ACE) of the two areas during simulation time T as shown in (5) as each area has ACE which is an indication of the frequency deviation in each area and also the tie line frequency deviation^18–20^. Equation (6) express ACE for certain areas

5 $$\:\mathrm{M}\mathrm{i}\mathrm{n}\mathrm{i}\mathrm{m}\mathrm{i}\mathrm{z}\mathrm{e}\:{\int\:}_{0}^{\mathrm{T}}({{\mathrm{A}\mathrm{C}\mathrm{E}}_{1}}^{2}+\:{{\mathrm{A}\mathrm{C}\mathrm{E}}_{2}}^{2})\:\mathrm{d}\mathrm{t}\:$$

6 $$\:{\mathrm{A}\mathrm{C}\mathrm{E}}_{i}=\:\sum\:_{j=1}^{N}\varDelta\:{P}_{ij}+{B}_{i}\varDelta\:{f}_{i}\:$$

where, ∆P_ij_ = the tie line power between area (i) and area (j), B_i_ = the frequency bias coefficient for area (i)^18^.

### Proposed cascaded controller

**Fig. 3:**
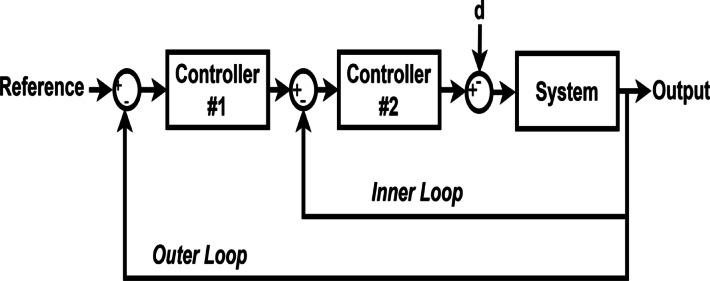
Block diagram of the cascaded control structure.

A cascaded control scheme is employed, combining the Adaptive Fractional-Order PI (AFOPI) controller and the PID controller to leverage the strengths of both controllers. Cascading control is a technique that improves system efficiency and responsiveness to disturbances by employing multiple controllers, thereby reducing downtime. In this scheme, the inner control loop is positioned closer to the disturbance, allowing for a faster response compared to the outer control loop, which improves overall system performance. The inner control loop utilizes the AFOPI controller, while the outer control loop employs a PID con- troller. The outer loop is primarily responsible for minimizing the area control error^20–23^. Figure 3 illustrates the block diagram of the cascaded control structure. The transfer function of the PID controller is given in Eq. (8), where Kp is proportional gain, Ki is integral gain and Kd is derivative gain. Inner loop controller 2 is AFOPI controller with gains K1, K2, K3, Kc and m, while outer loop controller 1 is PID Controller.

7 $${\rm{TF }} = {\rm{ Kp }} + {\rm{ Ki}} * ({\rm{1}}/{\rm{S}}){\rm{ }} + {\rm{ Kd}}*{\rm{S}}$$

## Simulation and results

In this paper, the proposed cascaded controller is subject to test its effectiveness in a single area power system and double area power system against different controllers. As, there are four case studies related to single area power system and another four case studies for the double area power system case studies.

The main parameters of both single area and double area systems are the governor model, turbine model, generator model, load model, droop (R), and governor frequency bias (B), that are represented in blocks as follows: Governor model = 1/(1 + STg), Turbine model = 1/(1 + STch), Generator and load model = 1/(MS + D), Droop = 1/R, and Governor frequency bias = B, D is expressed as a percentage load change and R is the speed regulation of the governor. M is the inertia constant as shown in Table 2. With the double area system is a two interconnected single area power system by tie-line. The load change in the single area affects only locally while in the double area the load change in one area affects both areas. Appendix 1 summarize the system model parameters for both single and double area power systems.

**Table 2:** System Parameters.

Parameters	Tch	Tg	*R*	B	D	M
**Single area**	0.3 s	0.1 s	0.05	21	1	10 s

Also in this section, the results will be evaluated in terms of key performance parameters, which are steady-state error, overshoot, undershoot, and settling time. As the system will be subjected to 0.1 p.u. load change at step size of 10 s. These parameters serve as critical indicators of the controller’s response. The results are expressed in delta frequency ∆F, providing a clear measure of system performance under different operating conditions.

### Single area power system

This section discusses in detail the single area power system parameters and the four different case studies, the first case is a single power system without time delay nor a wind disturbance. The second case study is similar to the first case but adding time delay. The third case study is the same as the first case study but adding wind energy as a disturbance and no time delay. While the fourth case study is similar to the third case study but adding the time delay of 2 s. Table 1 illustrates the single area system parameters.

#### Single area system without wind disturbance or time delay

**Fig. 4:**
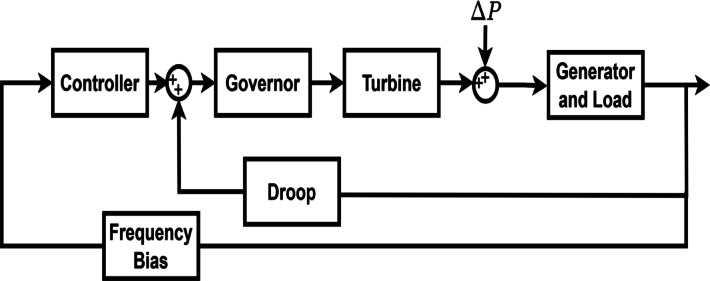
Case study 1 model.

**Fig. 5:**
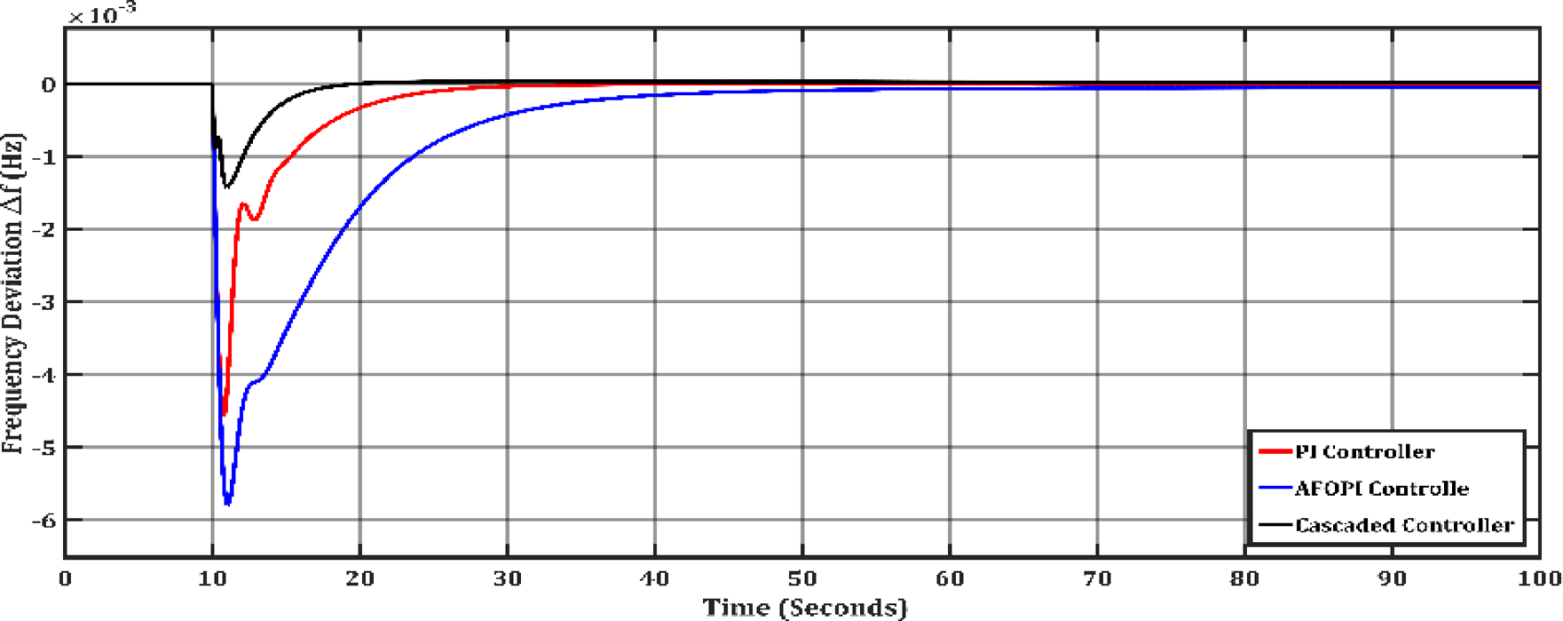
Delta frequency between three controllers for case study 1.

This single-area system is the simplest configuration among various power system models. The system has been reconstructed using the MATLAB Simulink program as shown in Fig. 4 with all system parameters without the time delay. Tables 3 and 4 shows the gains of PI HS based controller^16^ and AFOPI HS based controller^17^. Table 5; Fig. 5 demonstrate that the proposed cascaded controller significantly outperforms the other controllers. The overshoot is notably lower with the cascaded con- troller as it reached approximately − 1.2 × 10-3 p.u. which is equivalent to 49.94 Hz compared to the PI controller, that reached − 4.3 × 10-3 p.u. that is 49.77 Hz, and AFOPI controller dropped to −5.8 × 10-3 p.u. which is 49.72 Hz, the lowest among the three controllers. Additionally, the cascaded controller achieves steady-state in approximately 15 s, which is 9 s faster than the PI controller, which reaches steady-state in 24 s. These results indicate that the cascaded controller provides superior performance compared to the other two controllers.

**Table 3:** PI HS based controller gains of single area power system.

Gains	Kp	Ki
PI Controller HS Based^16^	0.45	0.32

**Table 4:** AFOPI HS based controller initial gains of single area power system.

Initial Gains	K1	K2	K3	Kc	m
AFOPI HS Based Controller^17^	1.98	1.98	0.46	1.59	0.96

**Table 5:** Proposed Controller initial Gains of single area power system.

Initial Gains	K1	K2	K3	Kc	m	Kp (PID)	Ki (PID)	Kd (PID)
Proposed Cascaded Controller	1.98	1.98	0.46	1.59	0.96	3	0.05	5

#### Single area system with time delay and without wind disturbance

**Fig. 6:**
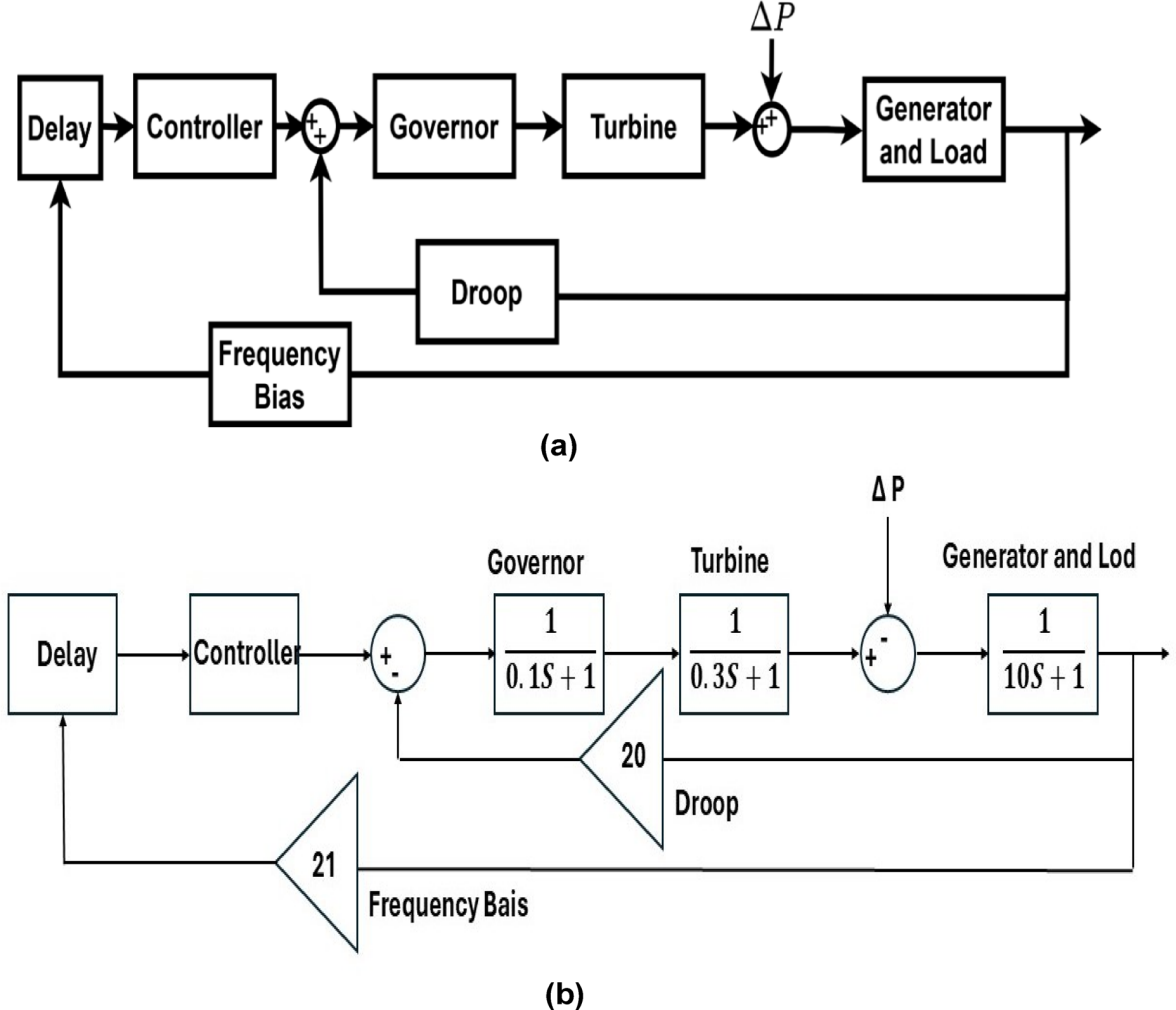
**a**: Model of Single area system without wind generator disturbance and with time delay. **b**: Transfer function-based model of Single area system.

This case study is similar to case study 1 but adding time delay to the system. The delay challenge to demonstrate the effectiveness of the proposed controller as the time delay is modeled as an exponential function with a time constant (2 s) where time delay is used to assess the response of the controller equipped with advanced technology designed specially to deal with the time-varying delays for the delay-dependent stability of LFC scheme as explained in^39^. Two additional tests are performed by setting the time delay to be 4 s and 6 s. Figure 6a and b represent the model with time delay and the transfer function-based model.

**Fig. 7:**
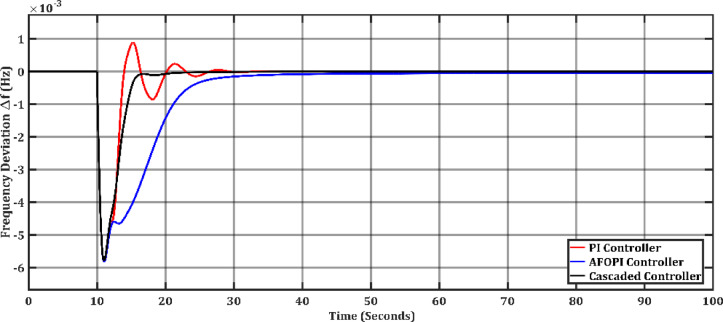
Delta frequency between three controllers for case study 2.

**Fig. 8:**
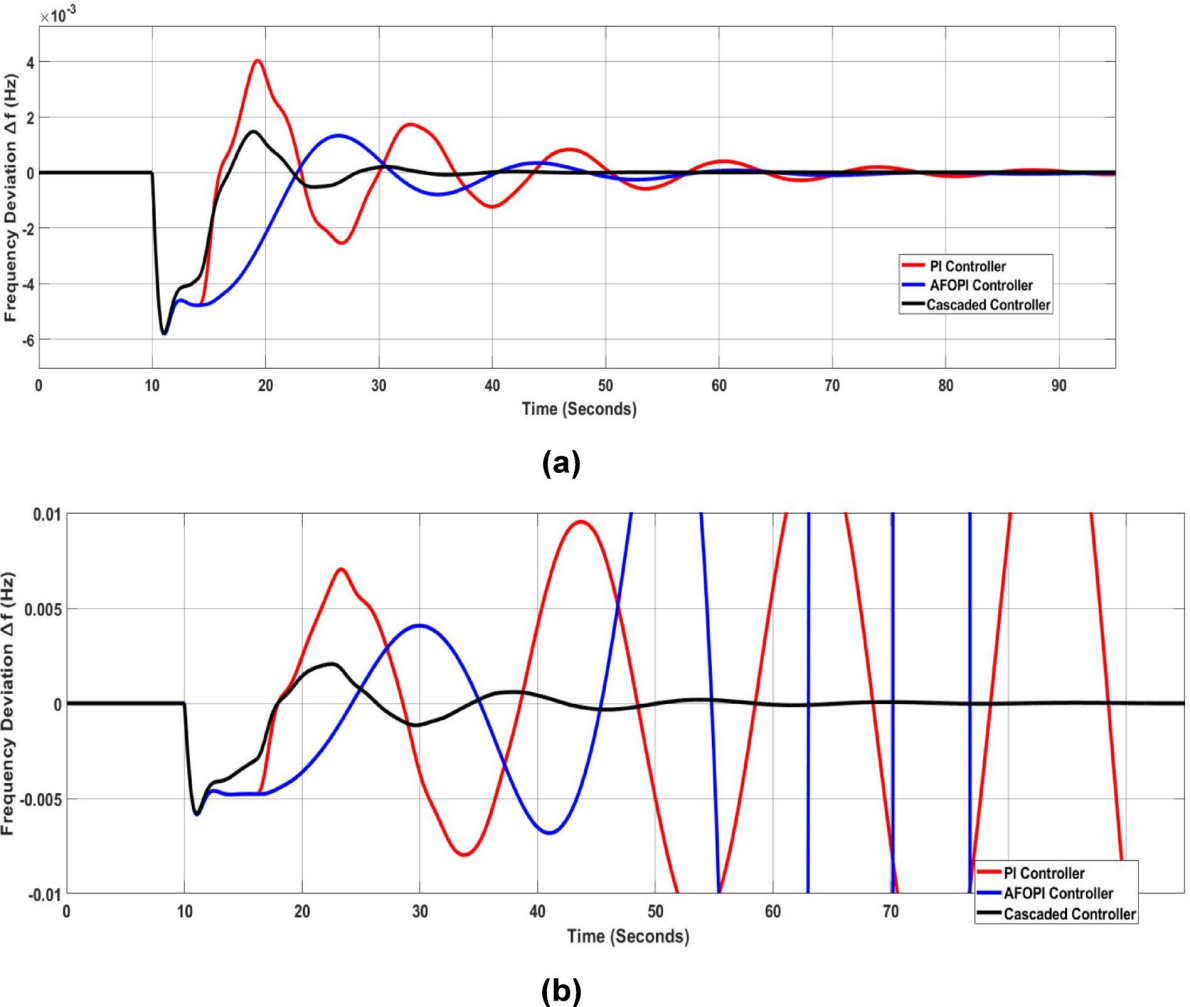
**a**: Delta frequency of 4 s time delay **b**: Delta frequency of 6 s time delay.

The system is subjected to three controllers: PI HS based controller, AFOPI HS based controller and the proposed cascaded controller. PI controller gains and AFOPI gains are compared with ref^16,17^ as shown in Tables 6 and 7. The results exhibit a high degree of similarity indicating that the simulation is robust and precise as shown in Fig. 7; Table 8 which represent the best fit gains. For the cascaded Controller, the initial parameters of AFOPI controller were obtained using HS algorithm and the gains of PID controller as they were obtained through trial and error directly. Figure 7 illustrates that the system was at steady state then the load change occurred and the response of the different controllers from which the cascaded controller outperforms both the PI and AFOPI controllers then the system returned to steady state again. Notably, the cascaded controller eliminates oscillations, and the settling time is significantly reduced from approximately 24 s to 15 s, reaching steady-state more efficiently. This indicates that the cascaded controller delivers excellent performance for this particular case study. For the additional validation tests, the model was subjected to increased time delays of 4 s and 6 s. The simulation results demonstrate that the proposed cascaded controller maintains superior dynamic performance compared to both the conventional PI controller and the AFOPI controller, confirming its robustness against significant time delays up to 6 s, as illustrated in Fig. 8a and b. Notably, when the time delay reaches 6 s, the system becomes unstable under the PI and AFOPI controllers, whereas the proposed cascaded controller preserves system stability and acceptable dynamic behavior.

**Table 6:** PI HS based controller gains of single area power system.

Gains	Kp	Ki
PI Controller HS Based^16^	0.45	0.32

**Table 7:** AFOPI HS based controller initial gains of single area power system.

Initial Gains	K1	K2	K3	Kc	m
AFOPI HS Based Controller^17^	1.98	1.98	0.46	1.59	0.96

**Table 8:** Proposed controller initial gains of single area power system.

Initial Gains	K1	K2	K3	Kc	m	Kp (PID)	Ki (PID)	Kd (PID)
Proposed Cascaded Controller	1.98	1.98	0.46	1.59	0.96	0.95	0.005	0.4

#### Single area system with wind disturbance

- The model of case study#3.

**Fig. 9:**
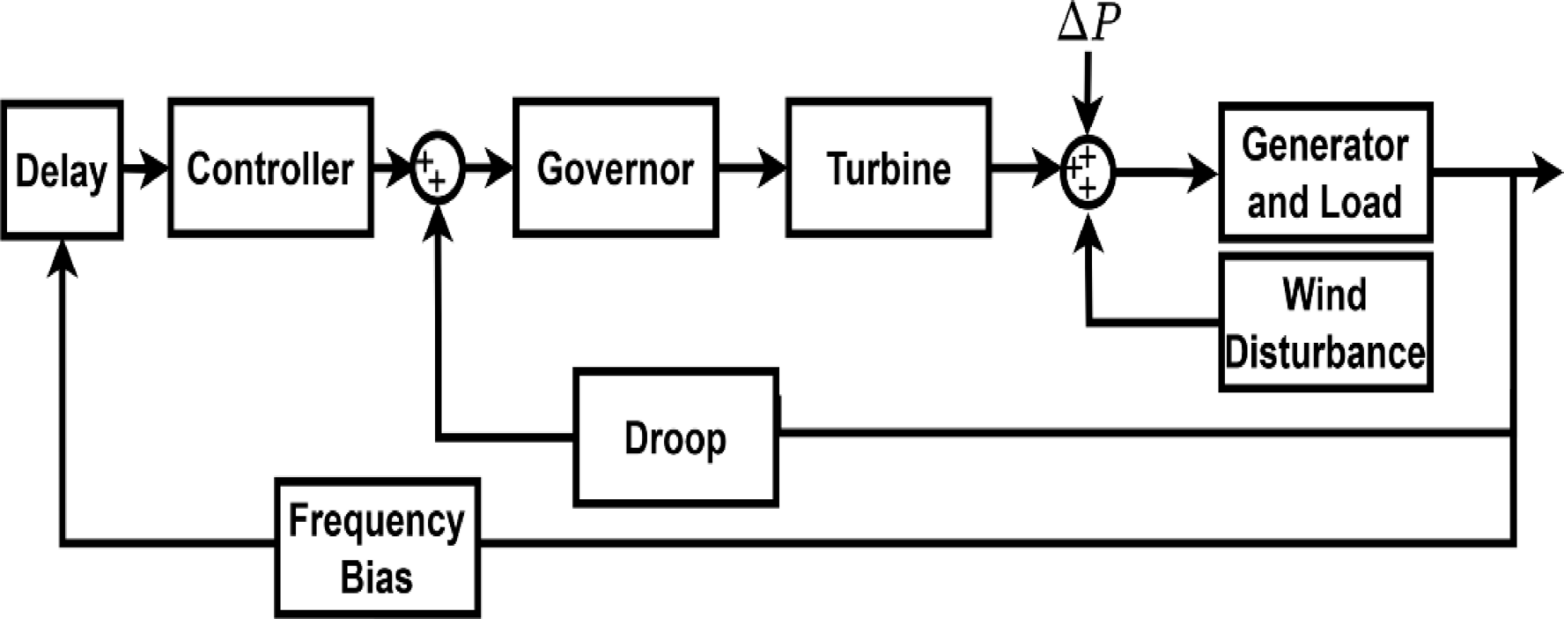
Model of Single area system with wind generator disturbance.

**Fig. 10:**
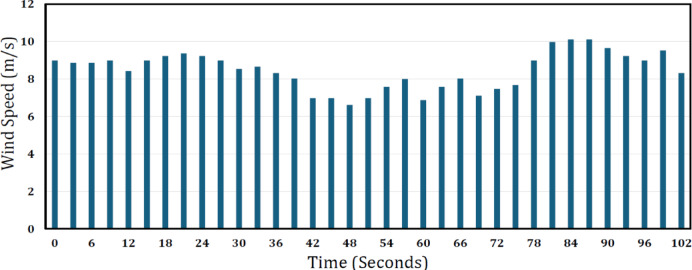
Variation of wind speed with time.

Case Study 3 is the same single area system with all system parameters but adding wind generators as disturbance. Figure 9 represents the system under study Wind generator data with swept area 5538.96 m2 and assume Cp = 0.5. the power of wind can be calculated as follows using (8). The power of wind shall be an input same as delta power as shown in Fig. 9, thus two inputs shall affect the frequency and the proposed controller shall be able to maintain the frequency within specific range and frequency deviation shall not be of major deviation

8 $$\:\mathrm{P}\mathrm{o}\mathrm{w}\mathrm{e}\mathrm{r}\:=\:\frac{1}{2}\:\mathrm{*}\:\rho\:\mathrm{*}\:\mathrm{C}\mathrm{p}\:\mathrm{*}\:\mathrm{A}\:\mathrm{*}\:\mathrm{V}3\:$$

where, ρ = density of air 1.225 (kg/m3), Cp = power coefficient, A = swept area (m2), V = wind speed (m/s) which are presented in Fig. 10 as^16^.

b) Results of case study#3.

**Fig. 11:**
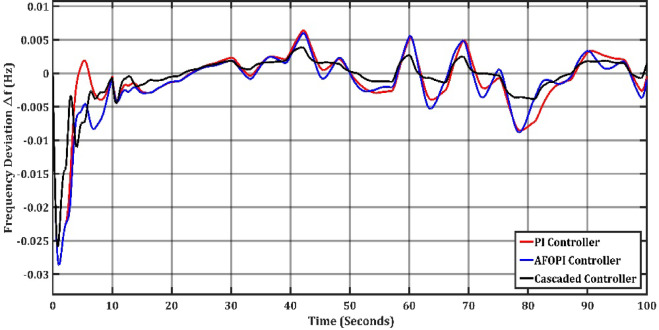
Delta frequency between three controllers for case study 3.

Tables 9 and 10 illustrate the gains of PI HS based controller^16^ and AFOPI HS based controller^17^. While Table 11; Fig. 11 demonstrates that the proposed cascaded controller performs better and more robustly than the other two controllers when subjected to wind disturbances. The cascaded controller experienced a significantly smaller undershoot, dropping only to −0.025 p.u. which is equivalent to 48.7 Hz, whereas the PI and AFOPI controllers dropped below − 0.026 p.u. that is equal to 48.6 Hz. Additionally, the cascaded controller exhibited reduced oscillations with lower peak amplitudes and achieved steady state faster than both the PI and AFOPI controllers. These results confirm the superior capability of the cascaded controller in handling renewable-related disturbances.

**Table 9:** PI HS based controller gains of single area power system.

Gains	Kp	Ki
PI Controller HS Based^16^	0.41	0.28

**Table 10:** AFOPI HS based controller initial gains of single area power system.

Initial Gains	K1	K2	K3	Kc	m
AFOPI HS Based Controller^17^	0.52	0.38	0.99	1	0.98

**Table 11:** Proposed controller initial gains of single area power system.

Initial Gains	K1	K2	K3	Kc	m	Kp (PID)	Ki (PID)	Kd (PID)
Proposed Cascaded Controller	0.52	0.38	0.99	1	0.98	0.4	0.01	0.3

#### Single area system with wind disturbance and no time delay

**Fig. 12:**
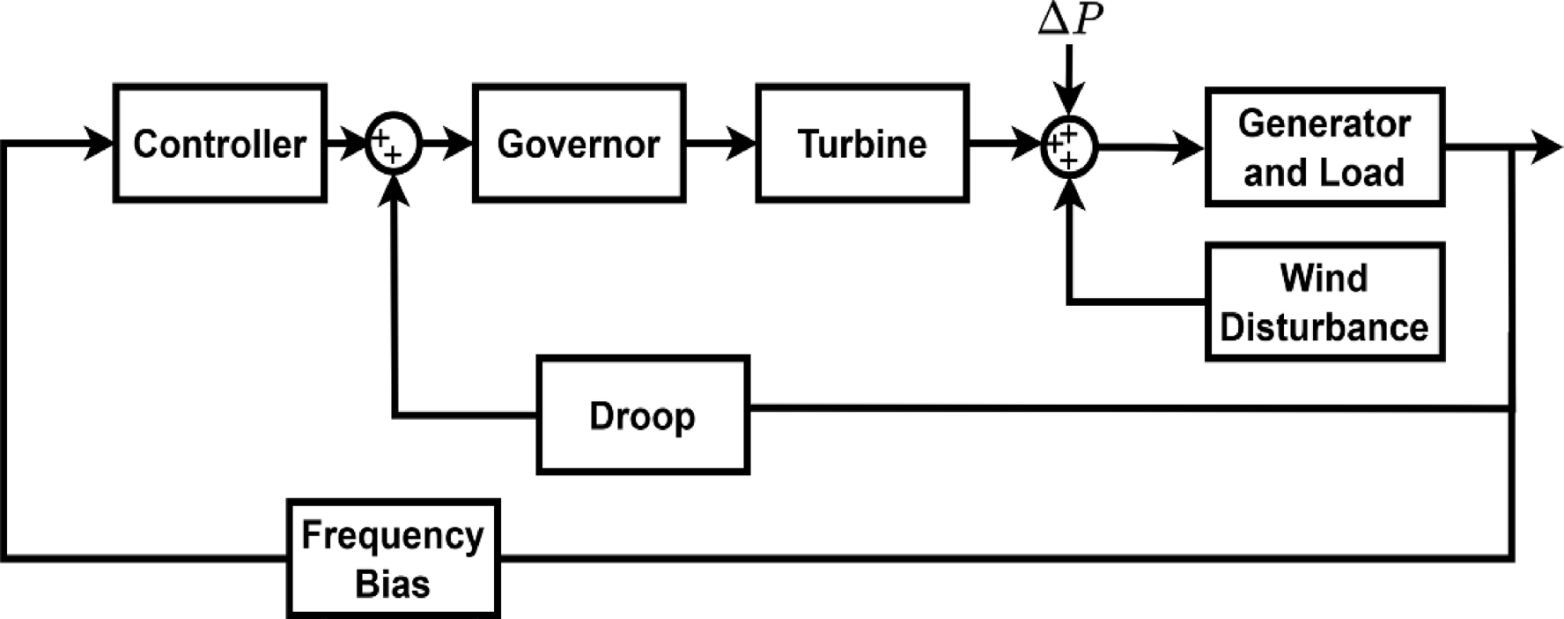
The model of case study 4.

**Fig. 13:**
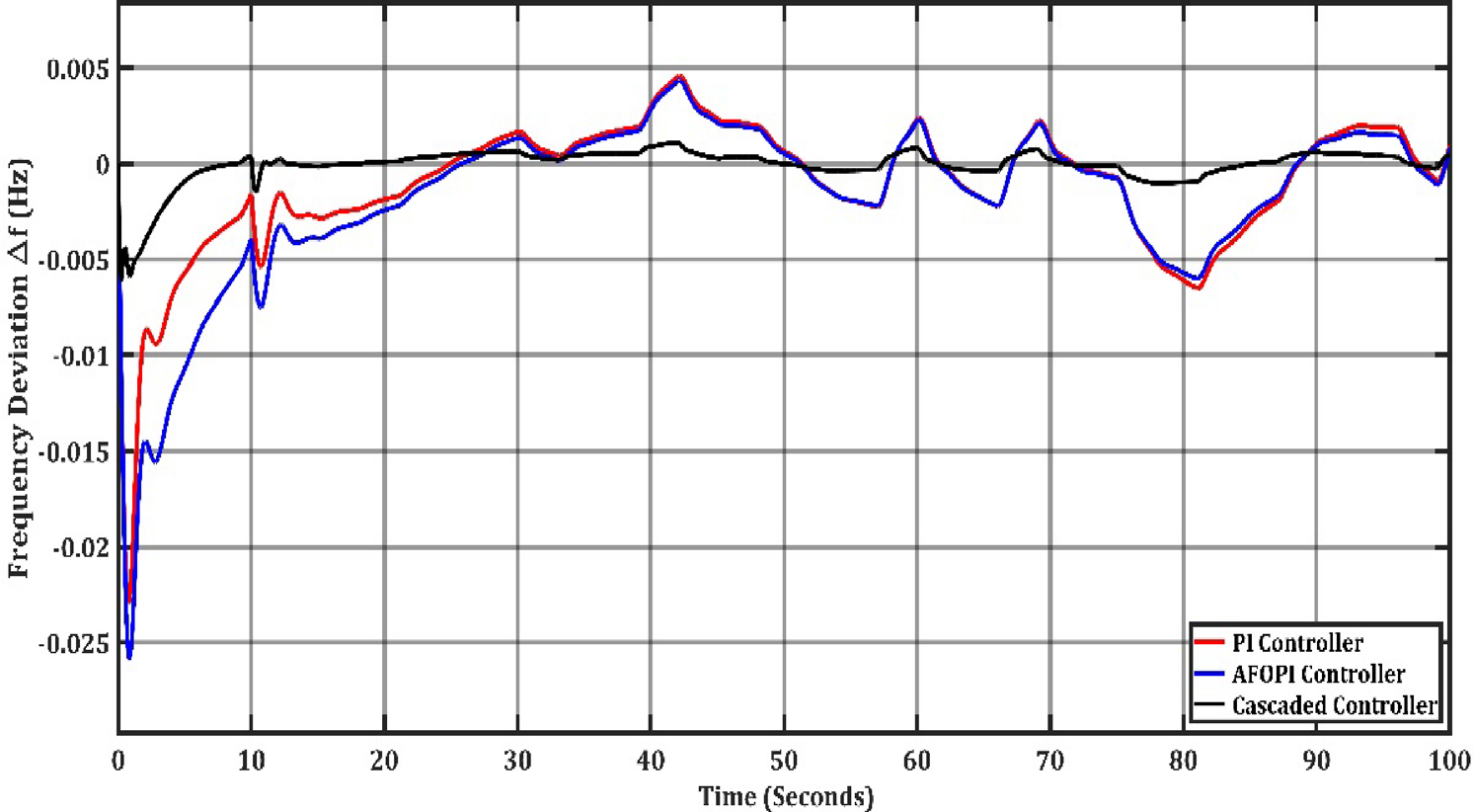
Delta frequency between three controllers for case study 4.

This is case study 4 which is identical to Case Study 3, except the time delay block has been removed. The model presented in Fig. 12 while Tables 12 and 13 illustrate the gains of PI HS based controller^16^ and AFOPI HS based controller^17^. The results are presented in Table 14; Fig. 13 clearly indicate that the cascaded controller delivers the best performance. The undershoot is significantly improved, dropping to −0.005 p.u., which is equivalent to 49.8 Hz, whereas the PI controller drops to.

−0.02 p.u., that is 48.85 Hz and the AFOPI controller drops below-0.02 p.u. Furthermore, the cascaded controller achieves steady-state in just 7.5 s—faster than the other controllers—and exhibits virtually no oscillations. These results confirm the enhanced stability and responsiveness of the cascaded control scheme.

**Table 12:** PI HS based Controller Gains of single area power system.

Gains	Kp	Ki
PI Controller HS Based^16^	0.41	0.28

**Table 13:** AFOPI HS based controller initial gains of single area power system.

Initial Gains	K1		K2	K3	Kc	m
AFOPI HS Based Controller^17^	0.52		0.38	0.99	1	0.98

**Table 14:** Proposed controller initial gains of single area power system.

Initial Gains	K1	K2	K3	Kc	m	Kp (PID)	Ki (PID)	Kd (PID)
Proposed Cascaded Controller	0.52	0.38	0.99	1	0.98	4	0.1	1

### Double area systems

**Fig. 14:**
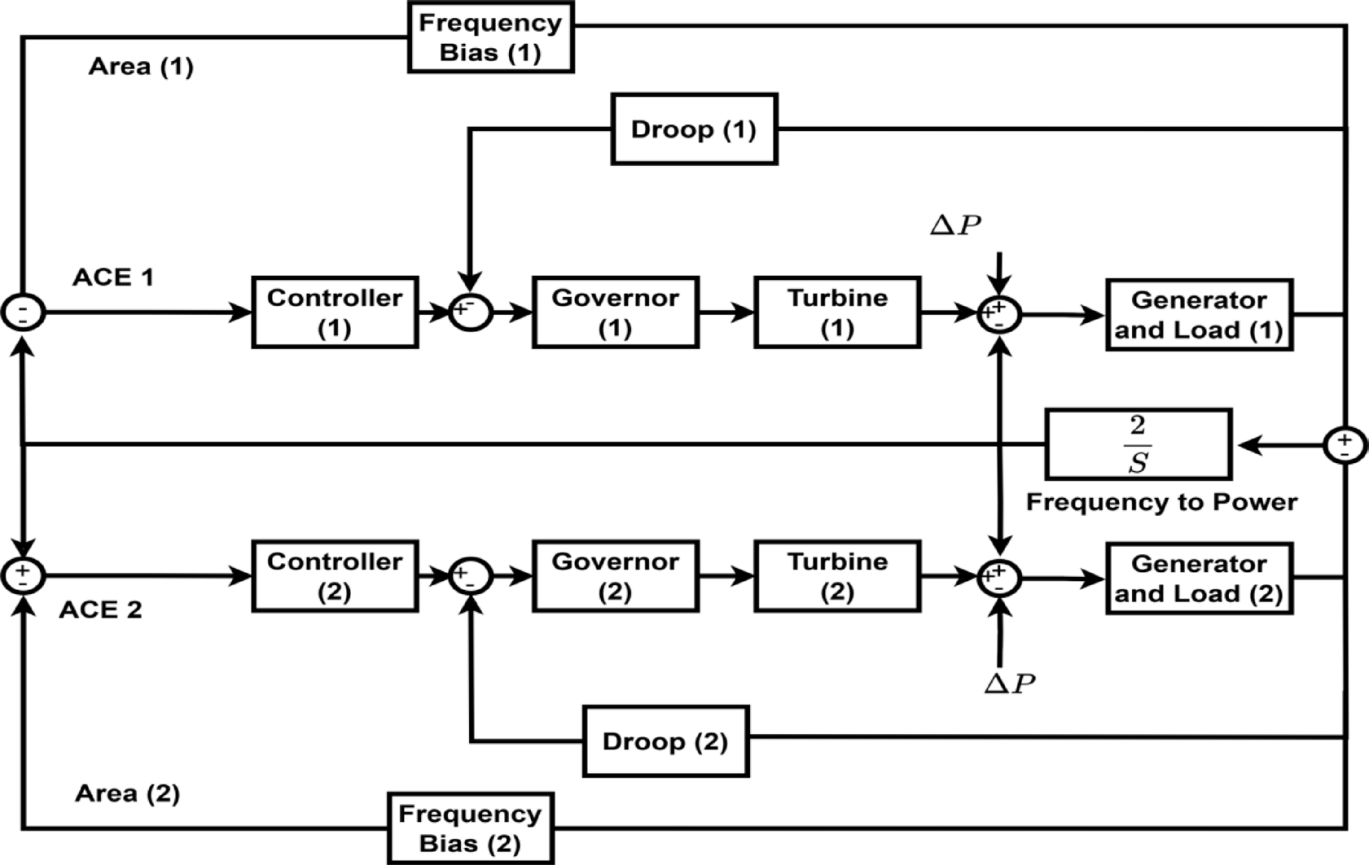
Model of Double area system.

**Fig. 15:**
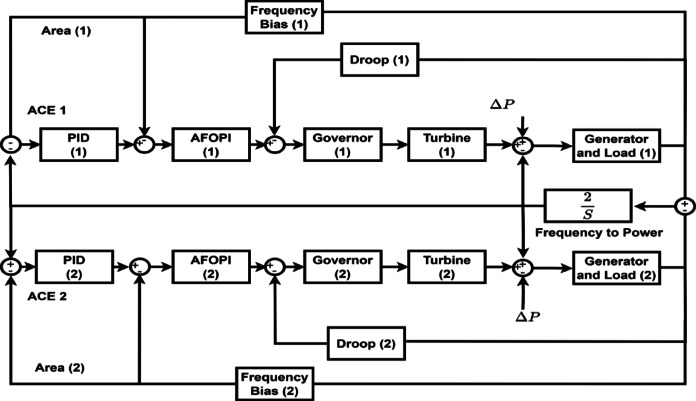
Model of double area system with proposed cascaded controller.

This section discusses the double area power systems which are dual single area power system that are linked together by tie-lines. Figure 14 represents the model of double area system. For double area, each single area has its own parameters as shown in Table 15^18^. Figure 15 shows the double area power systems with the proposed cascaded controller. The proposed controller output will be compared to PID HS Based controller^18^ output for the same load change. The time of the simulation will be 60 s in order to check the system’s steady state performance before, during and after the load change occurrence.

**Table 15:** Double area system parameters.

Parameter	T_ch_	T_g_	*R*	B	D	M
Area 1	0.5 s	0.2 s	0.05	20.6	0.6	10 s
Area 2	0.6 s	0.3 s	0.0625	16.9	0.9	8 s

#### Case study 1–1% load change at area 1

**Fig. 16:**
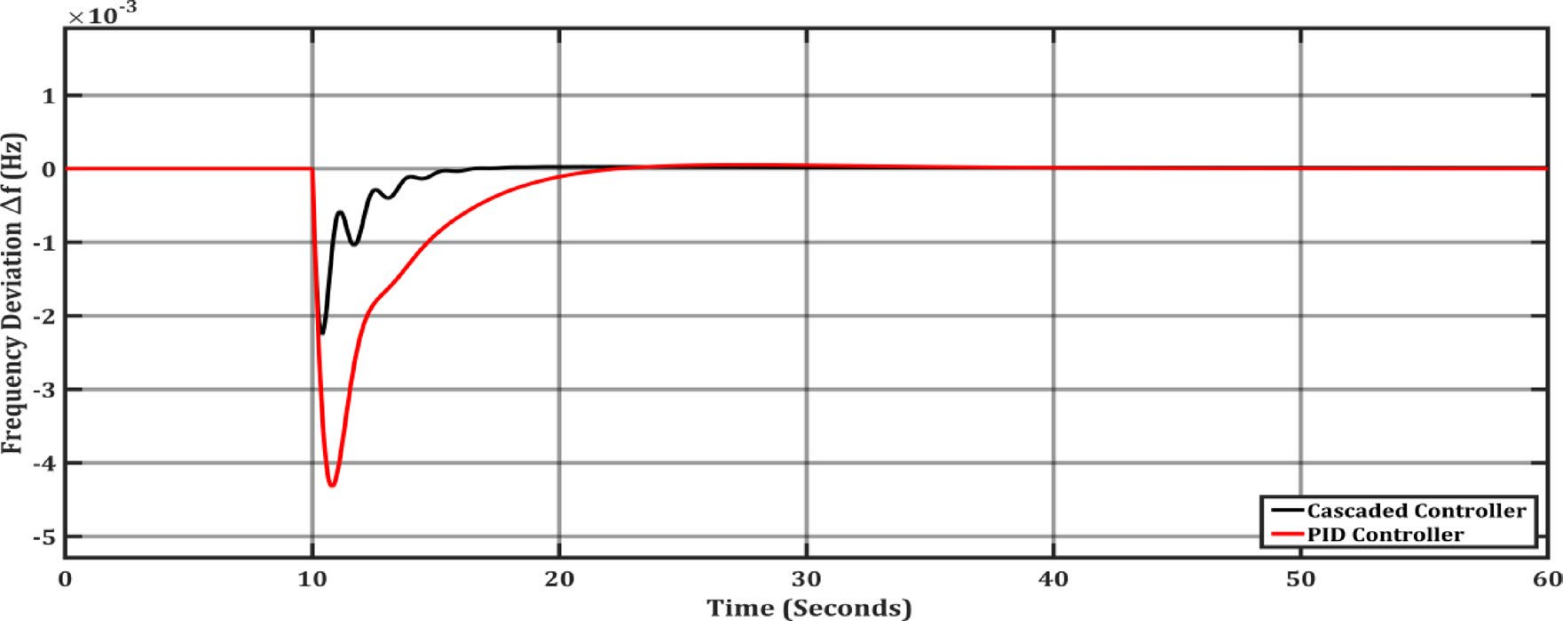
Frequency output of area 1 at 1% load change in area 1.

**Fig. 17:**
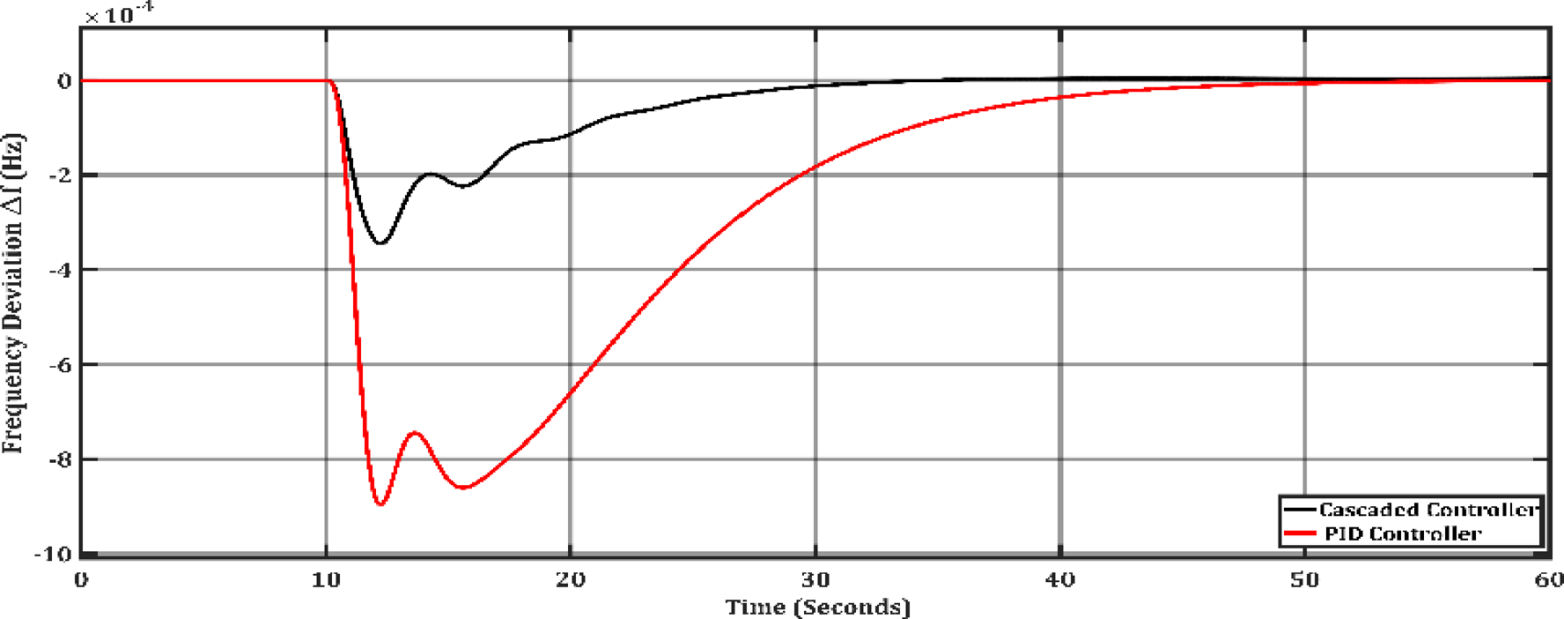
Frequency output of area 2 at 1% load change in area 1.

**Fig. 18:**
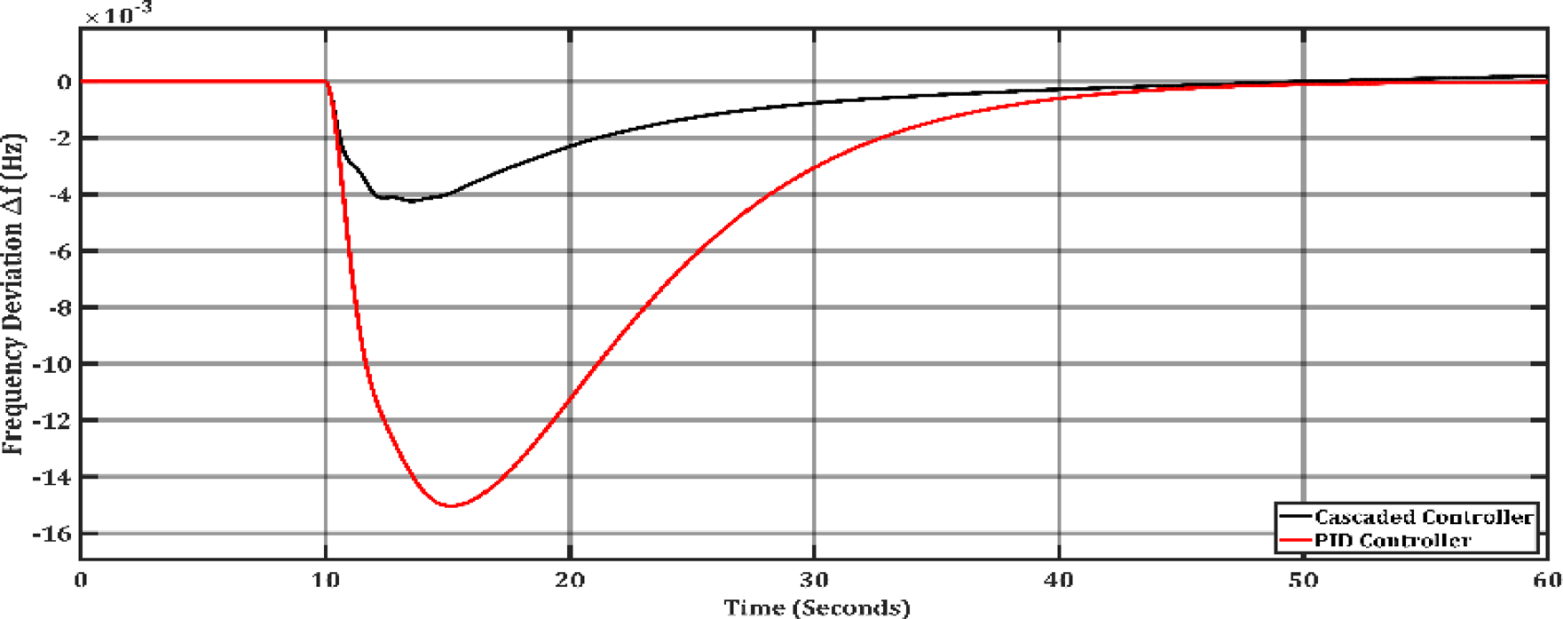
Frequency output of Tie-lines at 1% load change in area 1.

Case Study 1 of the double-area power system involves applying a 1% load change to Area 1 only, while Area 2 remains unaffected. Table 16 shows the gains obtained from PID HS based controller while the results for this scenario are summarized in Table 17. Figures 16 and 17, and 18 present the frequency responses of the cascaded controller in comparison with the PID HS based controller for Area 1, Area 2, and the tie-line, respectively from^18^. The initial parameters of the cascaded controller were obtained by two methods, first optimization using the Harmony Search (HS) algorithm, and by trial and error.

**Table 16:** PID HS based controller gains of double area power system.

Gains	Kp (PID HS Based)^18^	Ki (PID HS Based)^18^	Kd (PID HS Based)^18^
Area 1	0.35	0.22	0.44
Area 2	0.28	0.7	0.48

**Table 17:** Proposed cascaded controller gains of double area power system.

Initial Gains of Proposed Controller	K1	K2	K3	Kc	M	Kp	Ki	Kd
Area 1	2.81	2.75	0.31	6.5	0.92	1	0.01	2.5
Area 2	1.26	3.5	0.1	7	1.5	3	0.01	2

#### Case study 2 − 1% load change at area 2

In case study 2, the load change occurred at area 2 instead of area 1. The optimal gain and initial parameters values for controllers are listed in Tables 18 and 19 for the PID controller and proposed cascaded controller.

**Fig. 19:**
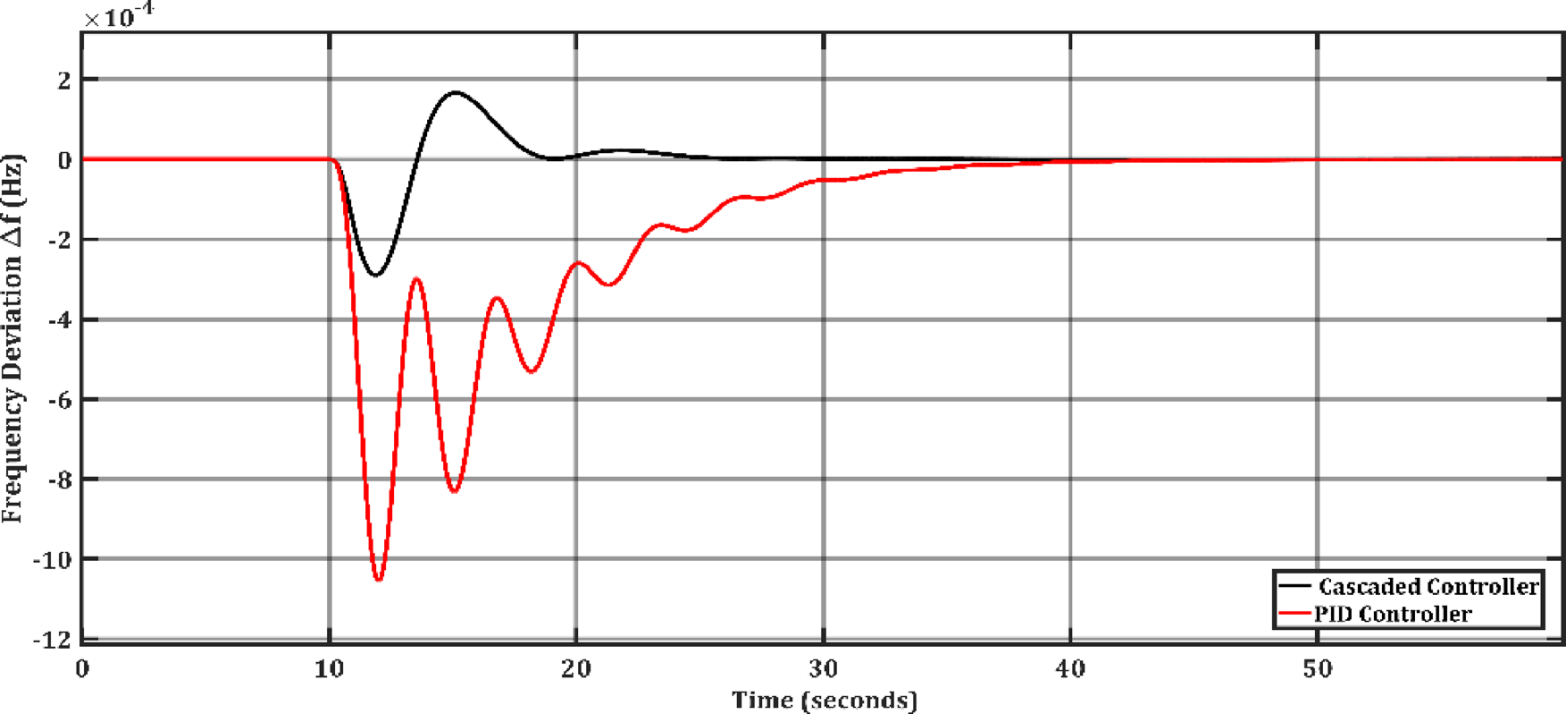
Delta frequency output of area 1 at 1% load change in area 2.

**Fig. 20:**
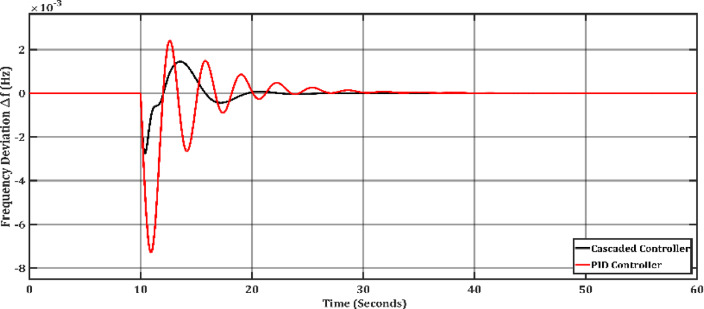
Delta frequency output of area 2 at 1% load change in area 2.

**Fig. 21:**
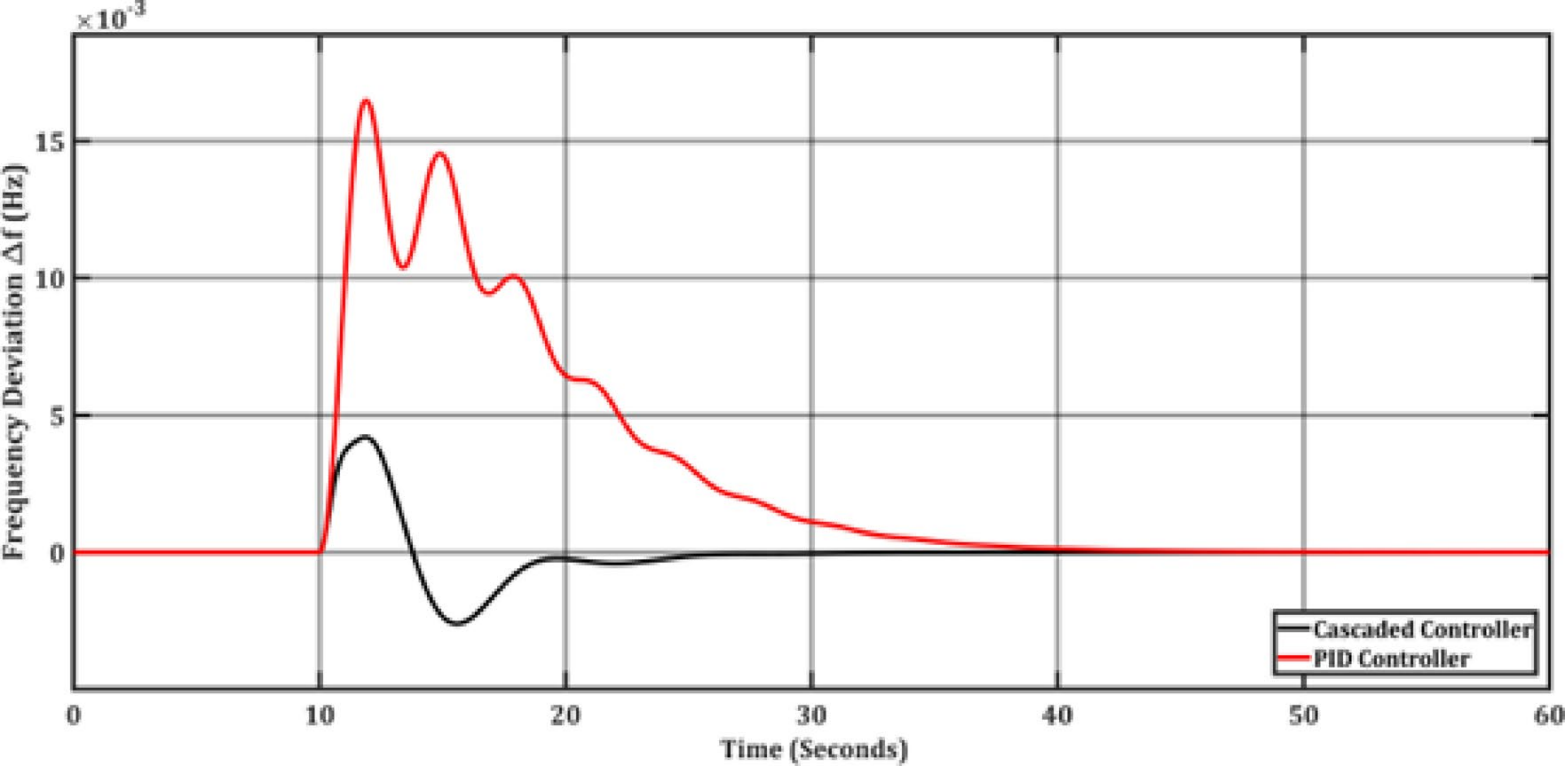
Delta frequency output of Tie-lines at 1% load change in area 2.

Figures 19 and 20, and 21 present the frequency responses of the cascaded controller compared to the PID controller. Figure 19 shows the frequency output in Area 1, where the cascaded controller achieves a settling time of approximately 7 s started from the load change occurrence, significantly faster than the 15 s required by the PID controller. Additionally, the cascaded controller exhibits minimal oscillations and an undershoot just below − 2 × 10-4 p.u. that is equal to 49.99 Hz, while the PID controller drops below − 10*10 − 4 p.u. that is 49.95 Hz. Figure 20 illustrates the frequency response in Area 2, where the cascaded controller achieves a faster settling time of 6 s with virtually no oscillations from beginning of the load change. The frequency undershoot remains above − 3 × 10-3 p.u. that is 49.9 Hz, and steady state is reached more quickly than with the PID controller. Finally, Fig. 21 shows the tie-line frequency response, confirming that the cascaded con- troller outperforms the PID controller in terms of reduced settling time, lower overshoot, and faster convergence to steady-state.

**Table 18:** PID HS based controller gains of double area power system.

Gains	Kp (PID HS Based)^18^	Ki (PID HS Based)^18^	Kd (PID HS Based)^18^
Area 1	0.61	0.3	0.28
Area 2	0.47	0.38	0.12

**Table 19:** Proposed cascaded controller gains of double area power system.

Initial Gains of Proposed Controller	K1	K2	K3	Kc	M	Kp	Ki	Kd
Area 1	1.43	2.11	0.24	1.47	1.3	1.5	2.5	1.1
Area 2	2.47	2.58	2.11	2.7	1.05	0.1	2.8	2.1

#### Case study 3- dynamic load change at area 1

**Fig. 22:**
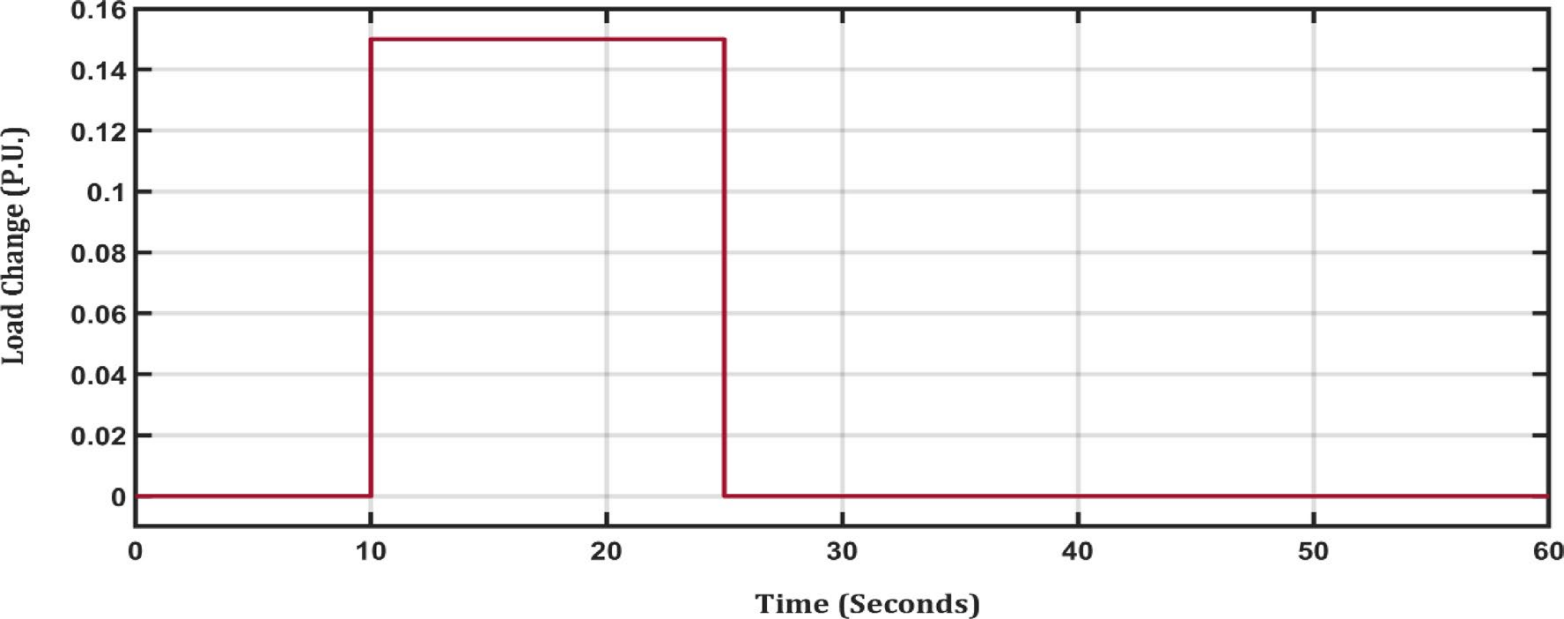
Dynamic load change.

Case Study 3 replicates the conditions of Case Study 1 but replaces the step load change of 0.1 p.u. with a dynamic load profile. The load change is zero from t = 0 s till t = 10 s then it increases by 15% at t = 10 s till t = 25 s then from t = 25 s till t = 60 s the load change returns to zero as illustrated in Fig. 22. The double area system will be tested with cascaded controller, PID controller and no control.

**Fig. 23:**
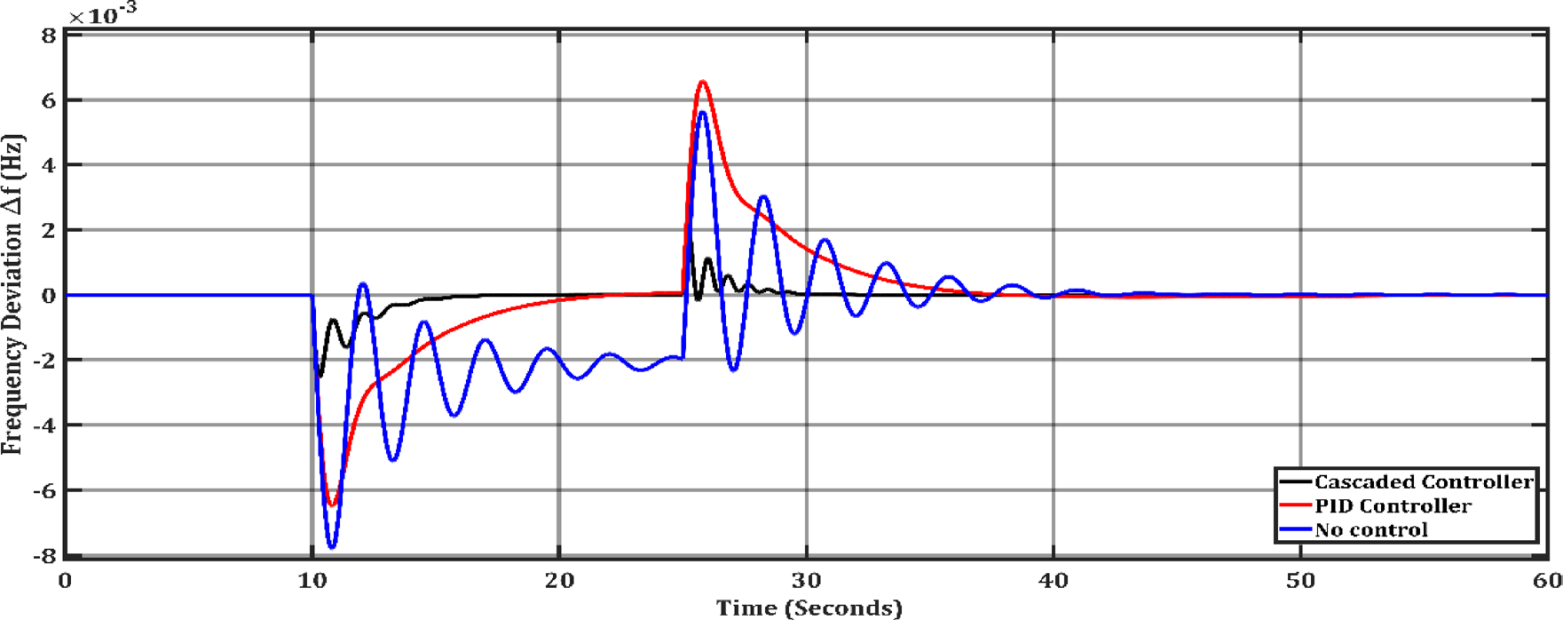
Delta frequency output of area 1 at dynamic load change in area 1.

**Fig. 24:**
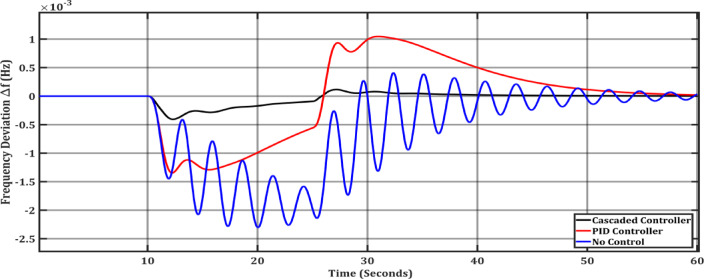
Delta frequency output of area 2 at dynamic load change in area 1.

**Fig. 25:**
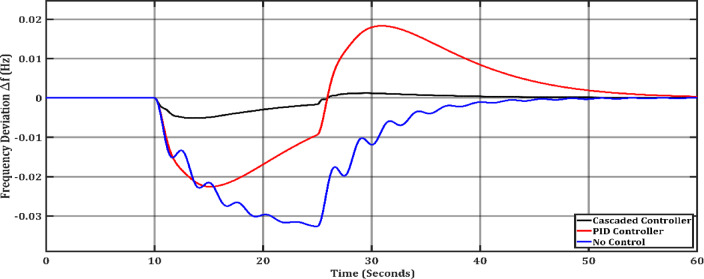
Delta frequency output of Tie-lines at dynamic load change in area 1.

Figure 23 demonstrates that the cascaded controller outperforms the PID controller in terms of faster settling time, lower overshoot and undershoot, and quicker achievement of steady state. Figure 24 further highlights the performance gap, showing that the cascaded controller maintains frequency close to zero delta frequency at almost 49.98 Hz, while the PID controller drops to −1.2*10-3p.u. that is equal to 49.92 Hz. Lastly, Fig. 25 confirms the superiority of the cascaded controller, with reduced undershoot, negligible oscillations, and a significantly more stable frequency response compared to the PID controller.

0.

#### Case study 4- dynamic load change at area 2

**Fig. 26:**
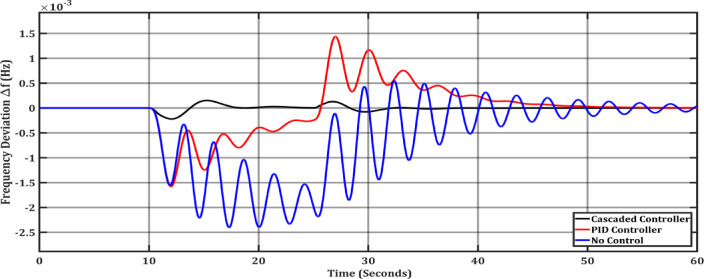
Delta frequency output of area 1 at dynamic load change in area 2.

**Fig. 27:**
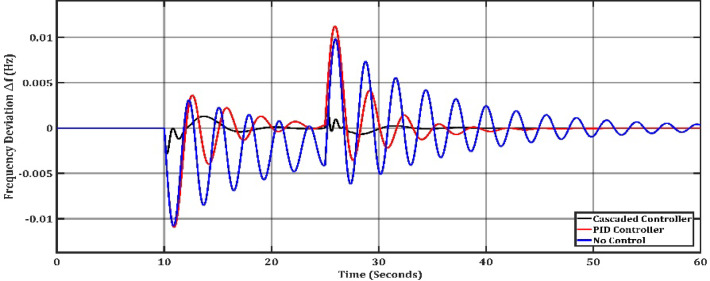
Delta frequency output of area 2 at dynamic load change in area 2.

**Fig. 28:**
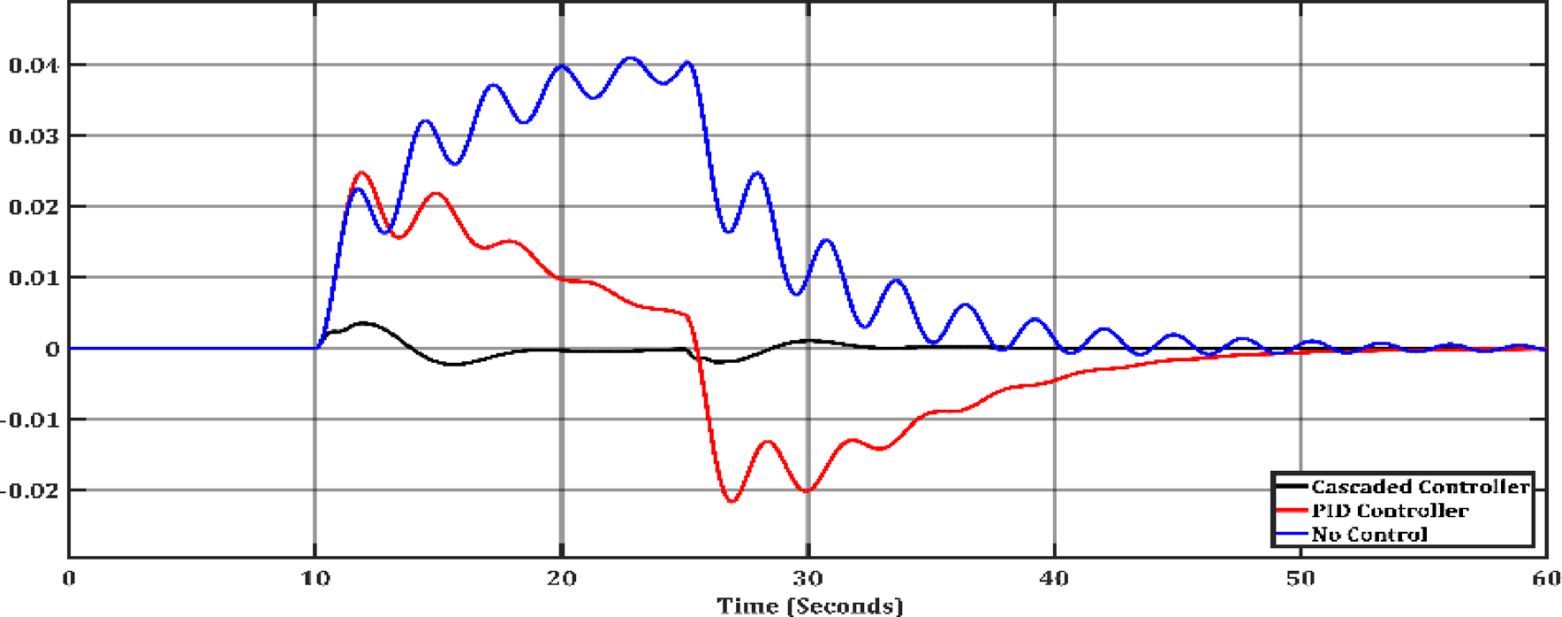
Delta frequency output of tie-lines at dynamic load change in area 2.

Figure 26 clearly demonstrates the superior performance of the cascaded controller, with the frequency output remaining nearly steady throughout the simulation, while the PID con- troller exhibits noticeable oscillations and struggles to reach steady state. Similarly, Fig. 27 reinforces this observation, showing that the cascaded controller achieves a more stable and responsive output compared to the PID controller. Finally, Fig. 28 further confirms the effectiveness of the cascaded controller, as it reaches steady state significantly faster, with minimal oscillations and a more stable frequency response than the PID controller.

## Conclusion and future works

This paper proposed a cascaded control structure that integrates an Adaptive Fractional- Order PI (AFOPI) controller with a classic PID controller to enhance dynamic performance and frequency regulation in both single-area and two-area power systems. The Simulation results across different case studies demonstrate that the proposed cascaded controller significantly improves system response by minimizing overshoot, reducing oscillations, and achieving faster settling times—outperforming traditional PI, standalone AFOPI, and PID controllers. As shown in the results, the cascaded controller decreases frequency deviation by 80% in Case Study 1 and reduces settling time by 25% in Case Study 2 while also shown effectiveness against different time delays. In Case Studies 3 and 4, the system frequency remains nearly constant at the nominal value of 50 Hz. For the two-area system, the controller reduces frequency undershoot by approximately 50% during a load change in Area 1 and achieves a 45–55% reduction in settling time across the two-area case studies. Furthermore, frequency remains nearly steady during dynamic load disturbances. The cascaded architecture leverages the adaptability of the AFOPI controller and the robustness and simplicity of the PID controller, providing effective control under various operating conditions, including disturbances and the integration of renewable energy sources. Future research may explore the application of advanced optimization algorithms to further fine-tune controller gains across different scenarios and system configurations. Overall, the proposed control scheme exhibits strong potential for use in both conventional and modern power systems with high levels of renewable energy penetration and observing the system for a longer period of time could reach a full day instead of seconds.

## Data Availability

The datasets used and/or analyzed during the current study are available from the corresponding author on reasonable request.

## Appendix 1 (see Tables 20, 21 and 22)

**Table 20:** -LFC components transfer functions.

Component	Transfer Function
**Governor**	$\:\frac{1}{\boldsymbol{S}{\boldsymbol{T}}_{\boldsymbol{g}}+1}$
**Turbine**	$\:\frac{1}{\boldsymbol{S}{\boldsymbol{T}}_{\boldsymbol{c}\boldsymbol{h}}+1}$
**Generator**	$\:\frac{1}{\boldsymbol{M}\boldsymbol{S}+\boldsymbol{D}}$
**Delay**	$\:{\boldsymbol{e}}^{-{\boldsymbol{S}\boldsymbol{T}}_{\boldsymbol{D}}}$
**Droop**	$\:\frac{1}{\boldsymbol{R}}$

**Table 21:** Parameters of LFC double area system.

Component	Parameter	Area 1	Area 2
**Governor**	$\:{\boldsymbol{T}}_{\boldsymbol{g}}$	0.2	0.3
**Turbine**	$\:\boldsymbol{T}\boldsymbol{c}\boldsymbol{h}$	0.5	0.6
**Generator & Load**	$\:\boldsymbol{M}$	10	8
	$\:\boldsymbol{D}$	0.6	0.9
	**R**	0.05	0.0625

**Table 22:** Parameters of LFC single area system.

Component	Parameter	Area
**Governor**	$\:{\boldsymbol{T}}_{\boldsymbol{g}}$	0.1
**Turbine**	$\:\boldsymbol{T}\boldsymbol{c}\boldsymbol{h}$	0.3
**Generator & Load**	$\:\boldsymbol{M}$	10
	$\:\boldsymbol{D}$	1
	**R**	0.05
**Delay**	$\:{\boldsymbol{T}}_{\boldsymbol{D}}$	2,4,6 s
